# Treatment of allograft renal cell carcinoma with partial nephrectomy in a pediatric kidney transplant

**DOI:** 10.1016/j.epsc.2021.102018

**Published:** 2021-08-30

**Authors:** Marina M. Tabbara, Mohamad Ammar Al Nuss, Jayanthi J. Chandar, Warren Alperstein, Gaetano Ciancio

**Affiliations:** a Department of Surgery, USA; b Pediatrics, Division of Pediatric Nephrology, USA; c Division of Hematology/Oncology, Sylvester Comprehensive Cancer Center, USA; d Urology, USA; e Miami Transplant Institute, USA; f University of Miami Miller School of Medicine, Jackson Memorial Hospital, Miami, FL, USA

**Keywords:** Renal cell carcinoma, Renal allograft, Pediatric transplant, de novo RCC, Case report

## Abstract

Renal cell carcinoma (RCC) is a common malignancy among kidney transplant recipients that often occurs in the native kidney. The incidence of RCC in the renal allograft is rare and carries the double risk of returning to dialysis and the development of metastatic cancer. The majority of reported cases of RCC in transplanted kidneys are in adult recipients and its occurrence in the pediatric age group is an uncommon event. There are currently no established guidelines on the treatment of RCC in transplant recipients.

We report our experience of a 15-year-old male who developed allograft RCC 12 years later after transplantation. MRI confirmed the presence of the mass near the hilum of the renal allograft and biopsy revealed a Papillary Renal Cell Carcinoma (PRCC) type I. A partial allograft nephrectomy was successfully performed with negative tumor margins. The patient’s serum creatinine 12 months post-operation was 1.9 mg/dL and presently he has no evidence of residual disease, recurrence, or metastasis.

Partial nephrectomy is an effective treatment option for renal allograft RCC as it spares the patient from returning to dialysis until retransplantation is possible and necessary.

## 1. Introduction

Renal cell carcinoma (RCC) is the most common type of kidney cancer in adults and occurs more frequently in the native kidney [1]. It rarely occurs in the renal allograft, with few cases sporadically reported with an incidence ranging from 0.18% to 0.5% [2,3]. RCC in transplant recipients can either be transmitted by the donor or develop de novo post-transplant [4]. It is usually detected in the early stage when the tumor diameter is less than 3 cm due to frequent follow-up and monitoring of the kidney transplant patient [5]. Surgical management of allograft RCC has been shown to include nephrectomy, partial nephrectomy, or radiofrequency ablation [6].

Majority of allograft RCC occur in adult recipients [3] and has been shown to be very uncommon in the pediatric kidney transplant population, with only 12 reported cases over the last 20 years [4]. We present our experience of a pediatric kidney recipient who developed an allograft renal allograft tumor 12 years after his transplant.

## 2. Case presentation

The patient is a 15-year-old male with a history of posterior urethral valve (PUV) whose renal function deteriorated at 18 months of age. Nephrostomy tubes were placed to determine if renal function would improve, however it continued to decline, and he ultimately required renal replacement therapy. He was placed on hemodialysis for 18 months prior to his transplant from a deceased donor at the age of 3. Unfortunately, information regarding the deceased donor was difficult to obtain. Post-transplant period was complicated by diarrhea, CMV, and EBV replication. He stabilized one-year post-transplant and was maintained on tacrolimus and prednisone for maintenance immune suppression.

**Fig. 1.**
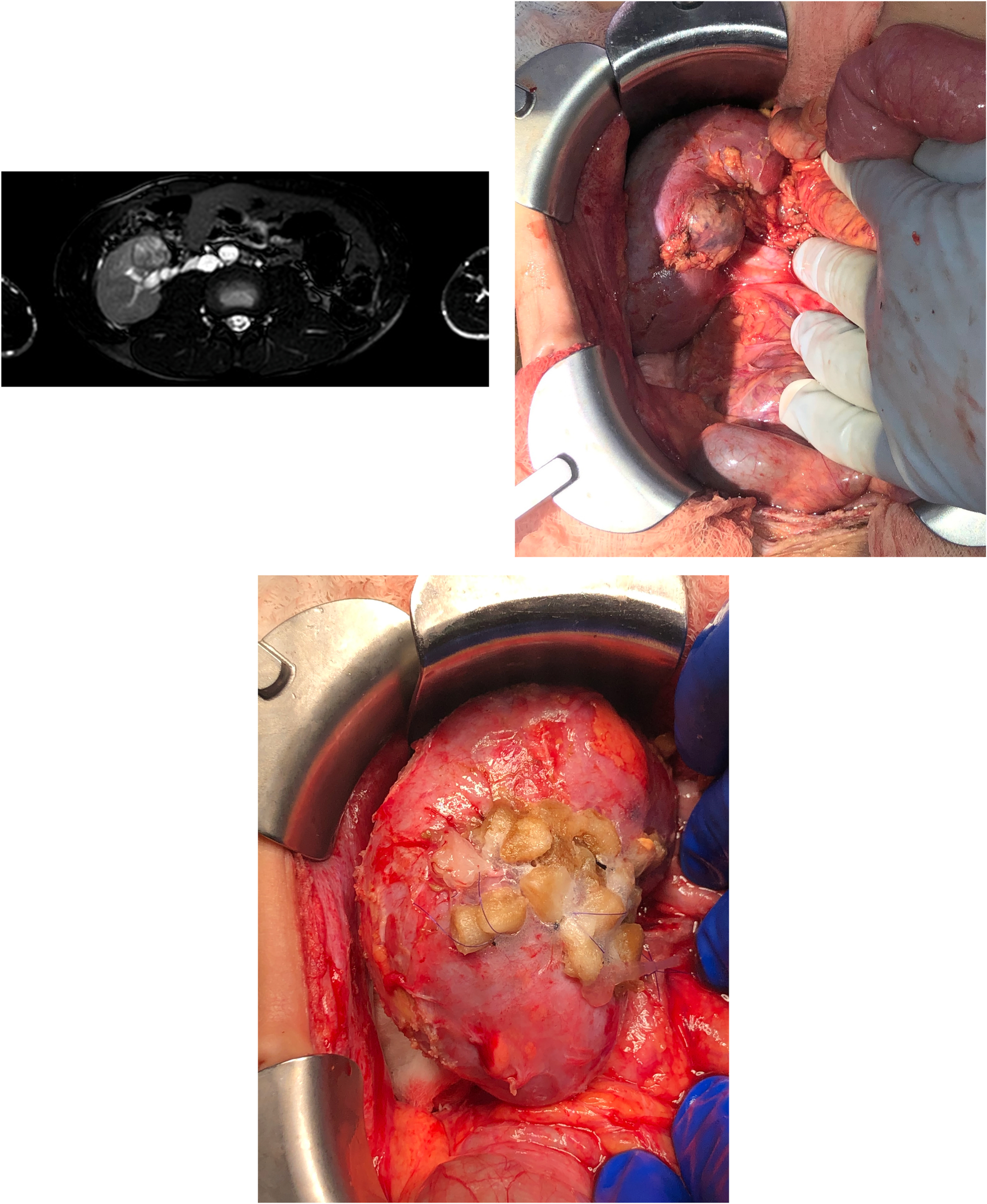
A, MRI of the abdomen showing the tumor in the allograft measuring 2.5 cm in diameter near the hilum and in close proximity to the collecting system; B, Intraoperative image showing the tumor close to the hilum and collecting system; C, Intraoperative image showing the kidney after performing partial nephrectomy. The collecting system was reconstructed, and the boundaries of normal renal parenchyma were oversewn. The edges of the open kidney were approximated with pledgets.

The patient eventually moved to a different state and was doing well until the age of 15. On a routine renal ultrasound done by his nephrologist, a small mass was noted in the transplanted kidney. Subsequently, an MRI of the abdomen confirmed the presence of a tumor in the allograft measuring 2.5 cm in diameter near the hilum and the collecting system (Fig. 1A). The diagnostic possibilities were renal cell carcinoma versus post-transplant lymphoproliferative disease. Further imaging studies including computed tomography (CT) scan and positron emission tomography showed atrophic native kidneys and no evidence of abdominal or chest metastasis.

With the objective of preserving residual allograft function and to avoid dialysis, renal biopsy and radiofrequency ablation were considered. A ureteral stent was placed in anticipation a possible complication of ureteral injury after the procedure. A biopsy of the tumor was performed, but due to adhesions of the bowel with the allograft, radiofrequency ablation was abandoned. Tumor biopsy revealed a Papillary Renal Cell Carcinoma (PRCC) type I.

Extensive discussions with the family and the multidisciplinary team were had regarding the challenges of removing a tumor close to the hilum and the collecting system. A plan was made to perform a partial nephrectomy, and the patient and his family were aware of the risk of having to undergo an allograft nephrectomy and dialysis if unsuccessful.

Scar tissue and multiple adhesions including an adherent colon were dissected during surgery. Direct visualization revealed a circumscribed tumor close to the hilum and collecting system (Fig. 1B). After mobilizing the kidney, the renal artery and vein were clamped. The tumor, along with 5 mm rim of apparently normal tissue, was removed sharply with a tenotomy scissors. The collecting system was reconstructed, and the boundaries of normal renal parenchyma were oversewn with 5–0 PDS. The edges of the open kidney were approximated with U-stich using 4–0 PDS and pledgets (Fig. 1C). A ureteral stent was left in place and removed after 2 weeks.

Tumor pathology revealed that the tumor was multifocal consisting of 2 foci, 2.5 × 2.0 × 1.6 cm and 0.4 × 0.3 × 0.3 cm, respectively, and confirmed the diagnosis of type 1 PRCC grade 2 with negative margins. There was evidence of lymphovascular invasion within the tumor. There was no evidence of rejection with minimal interstitial fibrosis of the renal parenchyma outside the tumor margins. Donor chimerism analysis of tissue revealed moderate chimerism (76–80%).

Post-operatively, the patient had adequate urine output. His creatinine reached a peak of 3.1 mg/dL by post-operative day two and reached a nadir of 1.9 mg/dL 12 months post-operation. Sirolimus was introduced 8 weeks after surgery, and he currently takes 3 mg daily and has a trough level of 3.9 ng/mL. He is currently off steroids and takes 1 mg of tacrolimus twice/day with a trough level of 2.2 ng/mL. CT of the chest and MRI of the abdomen and pelvis were done eight months after surgery and revealed no evidence of residual disease, recurrence, or metastasis.

## 3. Discussion

Renal transplant recipients have up to a 100-fold increase risk of developing renal cell carcinoma compared to the general population [6]. Majority of RCCs in transplant patients involve the native kidney and rarely develop in the allograft kidney. The published literature reports the occurrence of allograft RCC to be mostly in adults, with only 12 reported cases of allograft RCC in the pediatric age group [4]. It is a rare but serious issue that puts recipients at risk of returning to dialysis and developing metastatic cancer [6]. With that said, there are currently no established guidelines on the treatment of RCC in transplant patients [2]. Our case of a 15-year-old who developed RCC 12 years after transplantation highlights the complexities in diagnosing and managing allograft RCC in the pediatric population.

Among the allograft RCC that have been reported, only 24 (14.1%) were symptomatic and were diagnosed during routine ultrasound [3]. It has been shown that RCC occurs in the donor kidney either as a de novo cancer or as a pre-existing occult neoplasm [6]. Several authors concluded that RCCs were probably already present in the parenchyma at the time of transplantation [7–9], which was most likely the case in our patient based on chimerism analysis of allograft tissue.

Surgical management of allograft RCC has been shown to include nephrectomy, partial nephrectomy, or radiofrequency ablation [6]. Open radical nephrectomy has been shown to provide excellent oncological and functional results [10,11], however it is important to consider the preservation of the transplanted kidney and the return to dialysis. Nephron-sparing surgery is another appropriate alternative to radical nephrectomy, showing both good oncological control and graft survival [12]. Modification of immunosuppression to m-TOR inhibitors is an adequate measure in such patients [13]. It is important to note, however, that open partial nephrectomy in patients with renal transplant has greater technical challenges for achieving vascular control due to the presence of adhesions of the renal hilum [14].

Percutaneous radiofrequency ablation (RFA) is a viable, less invasive with low morbidity nonoperative option for RCC in the renal allograft. It has the advantage of sparing the patient from significant risks associated with total or partial nephrectomy of the transplanted kidney, however, it requires close and prolonged follow-up [4]. RFA should not be considered complete unless evidence of complete tumor destruction is documented histologically [15]. Cornelis et al. reported 20 recipients with allograft RCC in whom percutaneous thermal ablation was effective and performed safely [16]. RFA of small size renal cell carcinomas is gaining acceptance as a primary treatment for patients unsuitable for surgery or as a nephron-sparing therapy for patients with a solitary kidney or with renal impairment [15,17,18]. In our case, the RCC was very close to the hilum and there was no window for ablation. Furthermore, the large bowel was adherent to the renal allograft.

Retransplantation could offer significant survival benefit for the recipient and should be considered [19]. The KDIGO Clinical Practice Guidelines suggest that the ideal timing of kidney transplantation after treatment for malignancy depends on the cancer type and stage on initial diagnosis [20]. For patients with native kidney cancer, no waiting time is necessary for incidentaloma (<3 cm). Recommended waiting time for early malignancy is 2 years, and for large, invasive cancer, it is 5 years [19,20]. There are, however, no guidelines available yet for kidney re-transplantation after graft nephrectomy due to graft cancer [19], Barrou et al. report a case of renal graft carcinoma transmitted by the donor four months post-transplant who was re-transplanted two years later [21]. Tydén et al. describe a re-transplantation seven months after allograft RCC nephrectomy [22].

Close surveillance monitoring with imaging studies must be performed after surgery for allograft RCC. The American Urological Guidelines for RCC in the general population suggest performing imaging studies at 6 months, and 1, 2, and 3 years after diagnosis [23].

## 4. Conclusions

Treatment of allograft RCC must be tailored to the individual patient. We show that partial nephrectomy of the transplanted kidney is an effective treatment option for localized RCC, especially when RFA is not feasible. This approach spares the patient from returning to dialysis until retransplantation is possible and necessary.
